# Art of Caring Model for Emergency Care Patients and Professionals

**DOI:** 10.1111/nup.70024

**Published:** 2025-03-26

**Authors:** Carina Elmqvist, Michaela Ivarsdotter, Anna Bratt

**Affiliations:** ^1^ Department of Health and Caring Sciences, Centre for Interprofessional Collaboration within Emergency care (CICE) Linneaus University Växjö Sweden; ^2^ Department of Research and Development Region Kronoberg Växjö Sweden; ^3^ Konstsmide Väckelsång Sweden; ^4^ Department of Psychology, Faculty of Health and Life Sciences Linnaeus University Växjö Sweden

**Keywords:** art, caring, emergency care, ethics, patients, professionals, reflection model

## Abstract

The Art of Caring model is developed from a general structure of the flow in the encounter between the injured patients and the different professionals within emergency care, in turn founded on four phenomenological essences, which encompass the experiences of patients, next of kin, and various professionals during the encounter at the scene of an accident and at the emergency department. The Art of Caring model represents a philosophical and theoretical rethinking of an ethical approach. It draws upon the works of the Danish philosopher Løgstrup, the French philosopher Levinas as well as selected aspects of Merleau Ponty. The Art of Caring model is illustrated by coppersmith and artist Michaela Ivarsdotter, further developed and reflected upon with Anna Bratt, a psychologist working according to the compassion‐focused tradition. The model is made to disclose and visualise the Art of Caring and facilitate reflections on achieving a win‐win situation for both patients and different professionals within emergency care. Healthcare involves a variety of professions, and for the benefit of the patient, we must recognise the significance of professionals taking on the advocacy role from a caring science perspective, which includes the unique and shared experiences of the lifeworld. This is a challenge within the context of demanding efficiency and time pressure in emergency care. To address this, a concrete action plan for ethical reflections is needed to find a balance between giving and receiving, essential for healthcare professionals to avoid compassion fatigue. In the context of ethical competence and the challenges faced by different healthcare professionals within emergency care, the Art of Caring model could be used for ethical reflections, as an approach to achieve a balance between patient advocacy, ethical considerations, and effective emergency care delivery. Achieving this goal will lead to better patient outcomes and a more supportive work environment for the entire emergency care team.

## 1 Introduction

The Art of Caring model is developed from the general structure of interactions between patients and the various professionals within emergency care (Elmqvist 2011, 2016). The model is founded on four phenomenological essences, which encompass the experiences of patients, their next of kin, and various professionals during encounters at the scene of an accident and at the emergency department (Elmqvist et al. 2008, 2010, 2011, 2012). The general structure of the encounter reveals the vulnerability of the patients seeking emergency care and how the professionals and the patient come close together in a mutual space during life‐saving actions. However, as the patient's condition stabilises, they find themselves in an interspace. For the patient this means an empty space with feelings of being interesting and at the same time uninteresting, a sort of a paradoxical realm of care. For professionals, this interspace offers a much‐needed breathing space, moreover it can lead to feelings of for instance moral distress, caught in an ethical dilemma, between the high demands of efficiency and time pressure and the patient's unmet existential needs and desire for a meaningful encounter (Elmqvist 2016). Within these interspaces, it becomes essential for professionals to take an advocacy‐role and meet the patients’ needs without risking compassion fatigue.

The Art of Caring model represents a philosophical and theoretical rethinking, drawing from ethical approaches by the Danish philosopher K.E. Løgstrup, (The Ethical Demand 1992), the French philosopher E. Levinas, (Ethics and Infinity 1993) and selected aspects of M. Merleau Ponty, (The Visible and The Invisible 1964/1992). The Art of Caring model is illustrated by coppersmith and artist Michaela Ivarsdotter, further developed and reflected upon with Anna Bratt, a psychologist working according to the compassion‐focused psychotherapy tradition. Through art illustrations the model is made to disclose and visualise the interspaces experienced by both patients and professionals. By looking at something as a figure against a background of something else, it becomes easier to reflect on and uncover the essence of care—the Art of Caring. This model facilitates reflections aimed at achieving a win‐win situation for patients and diverse professionals within emergency care.

## 2 The Art of Caring Model Within Emergency Care

**Figure 1:**
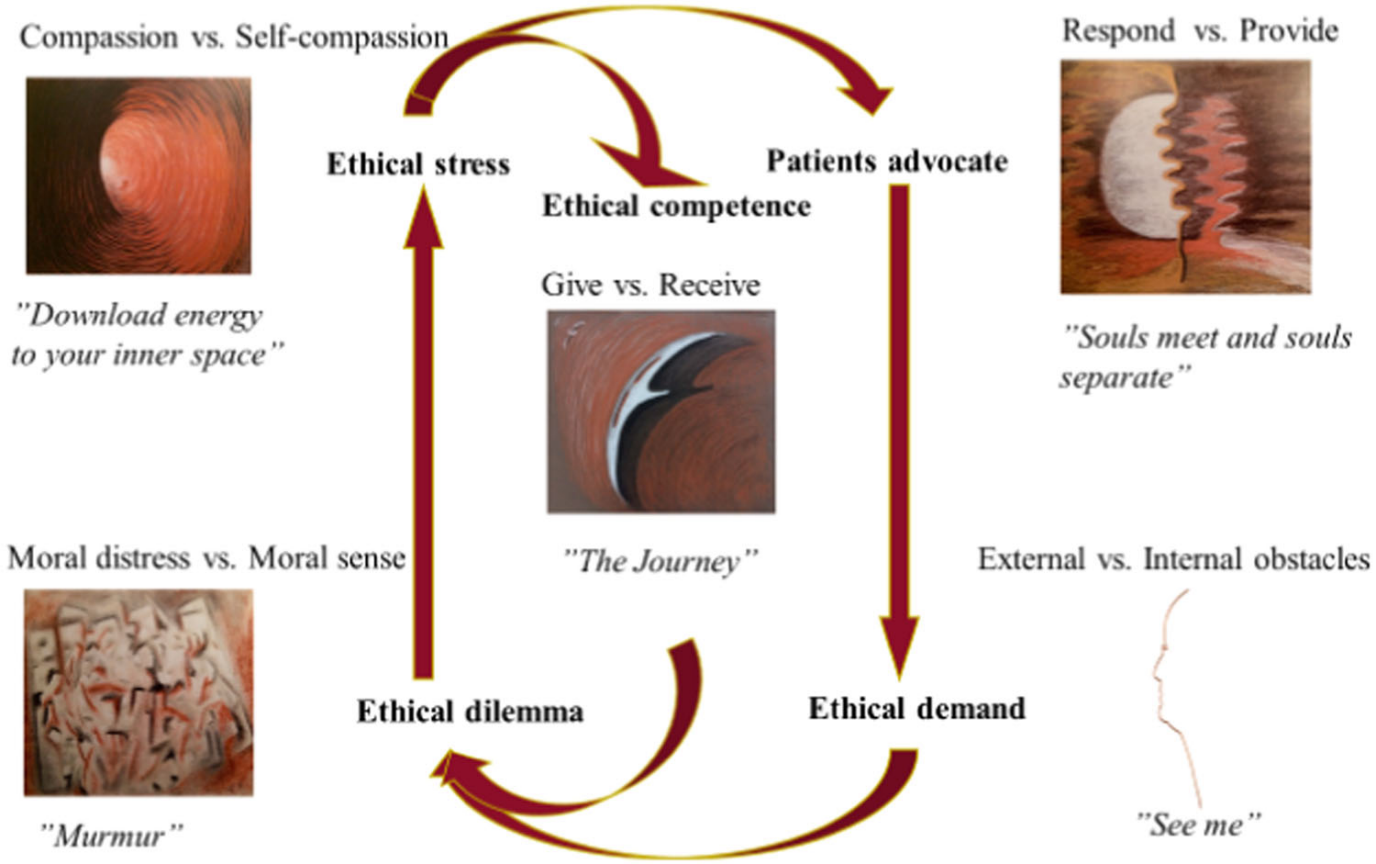
The Art of Caring model within emergency care.

As a patient advocate, it is crucial to be attuned to the others' lifeworld while providing care. Balancing this responsibility with responding to the patients' caring needs is essential. As a professional, you act based on ethical demands, but your inevitable face both external and internal obstacles during patient encounters. While you can learn to navigate inner challenges, managing external ones proves more difficult. Ethical dilemmas, such as balancing efficiency with patient needs, inevitably arise. Whether visible to others or not, acknowledged to oneself or not, these dilemmas impact professionals. Situations from work may linger, affecting you even after leaving the workplace, resulting in moral distress. Embrace these reflections and your moral sense as opportunities to enhance ethical competence. Remember moments that brought energy to all involved in different ways. Every small act of care contributes to nourishing your inner space, alleviating ethical stress, and fostering your role as a patient advocate. Prioritising professionals' well‐being is vital for compassionate care, as high levels of stress due to heavy workloads can lead to a disconnection from those under their care. Compassion training and reflective practice aimed at cultivating feelings of love and care towards oneself and others can reduce the risk of burnout and stress related health problems. Ethical competence is strengthened by engaging in both giving and receiving, as well as sharing experiences through spoken and unspoken communication. The courage to touch and be touched emotionally, creates a 'win‐win' situation for everyone involved (see Figure 1). Through an awareness of concepts such as respond versus provide, external versus internal obstacles, moral sense versus moral distress, compassion versus self‐compassion and give versus receive, the essence of care or the Art of Caring becomes visible. The Art of Caring model has been visualised through art, enabling a tangible representation of its essence and deepening the reflections.

## 3 Patient Advocate

Patient advocacy is an essential aspect of healthcare, especially within nursing. However, it encompasses diverse meanings and perspectives (Mitchell and Bournes 2000; Vaartio and Leino‐Kilpi 2005; Cole et al. 2014; Josse‐Eklund et al. 2014; Vitale et al. 2019; Abbasinia et al. 2020; Heck et al. 2022). Despite its less defined role (Cole et al. 2014), the theoretical understanding of human beings provides the foundation for action (Mitchell and Bournes 2000). In this context, we specifically refer to patient advocacy from the caring science perspective, which serves as a knowledge base for various healthcare professions (Dahlberg and Segesten 2010). Caring science emphasises the relationship between professionals and patients, aiming to understand the unique and shared subjective experiences of the lifeworld, which are unique for every person and at the same time shared with other (Bengtsson 1998; Dahlberg and Segesten 2010).

### 3.1 Respond Versus Provide

**Figure 2:**
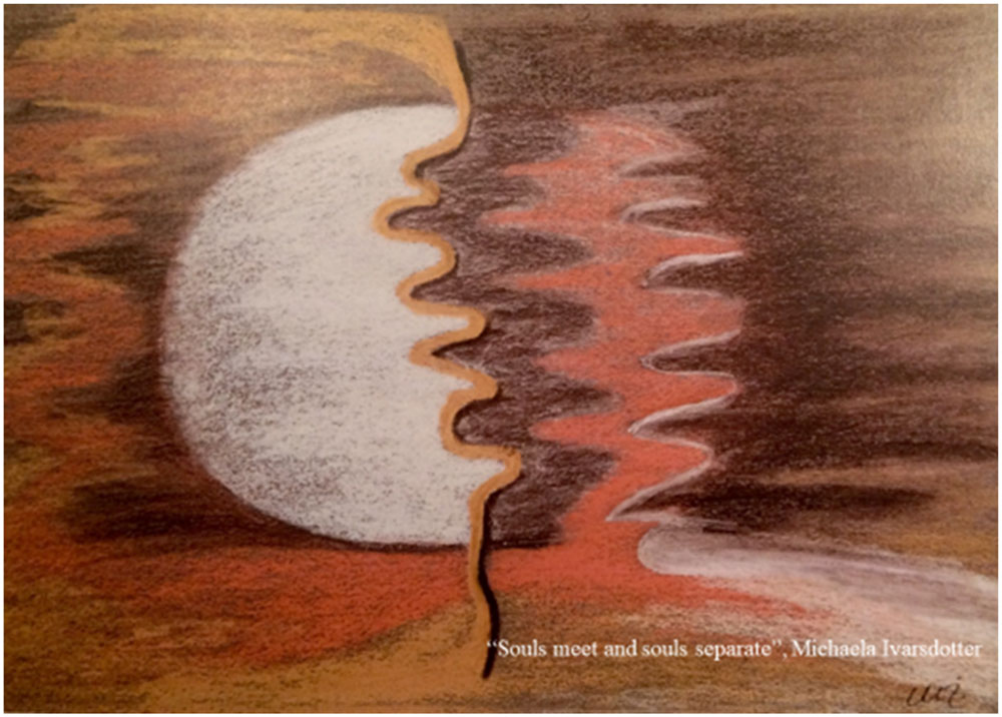
'Souls meet and souls separate'.

This piece of art, titled 'Souls Meet and Souls Separate' (Figure 2), symbolises the intersection of different lifeworlds, when meeting each other, each with unique experiences and expectations in their encounters (Elmqvist 2011). What expectations do patients have for first responders at the scene of an accident and for first providers at the Emergency Department (ED). Do they see themselves primarily as patients, or do they view their identity as having a body with a disease? The question arises: 'Who am I as a person now?'

Even though it is relieving for the patient to hand over responsibility for the body to responders, it is still important not to become too dependent on health‐care personnel. For the injured at the scene of an accident it means a dynamic movement between control and dependence, and between fear and security. The threat to life empowers the struggle for survival, which crucial to prevent resignation (Elmqvist et al. 2008). For patients at the ED, lack of control can make them feel like they are in an empty space, where understanding the past, present and future becomes important. They want to know what is happening, how, why, when and for how long (Elmqvist et al. 2011).

What kind of expectations do professionals have for the patient and themselves? How do they perceive themselves in the encounter with the patient? Are they working to respond to someone's caring needs or to provide care to those seeking it? Being the first responder at the scene of an accident entails a continuous movement between being and doing where everything is interconnected and understood in relation to each other simultaneously (Elmqvist et al. 2010). Similarly, being the first provider at an ED involves a continuous movement between providing care and responding to life‐saving demand, often leading to a delicate balance them in‐between (Elmqvist et al. 2012). With the above descriptions in mind, are there a distinction between being and doing ‐ providing and responding, when it comes to being the patients advocate and providing the needed support to the patient? Let's see if we can find more answers through ethical aspects within caring science.

## 4 Ethical Demand

Within caring science, the ethical aspects of the caring encounter form the basis for care (Eriksson 1995), this often involves an existential view of human relations, as discussed by Løgstrup and Levinas (Dahlberg and Segesten 2010; Eriksson 1995). According to Løgstrup, the ethical demand concerns how I should act, which means that someone else depends on how I act. Løgstrup uses a 'Hand' as a metaphor for holding a part of another person's life in my hand (Løgstrup 1992). Similarly, Levinas describes the ethical demand as the way another person's presence appeals to my sense of responsibility, a task that should be difficult to ignore. Levinas uses the 'Face' as a metaphor, signifying not only seeing the other person's physical face but also recognising their unique otherness and taking responsibility for them (Levinas 1993).

### 4.1 External Versus Internal Obstacles

**Figure 3:**
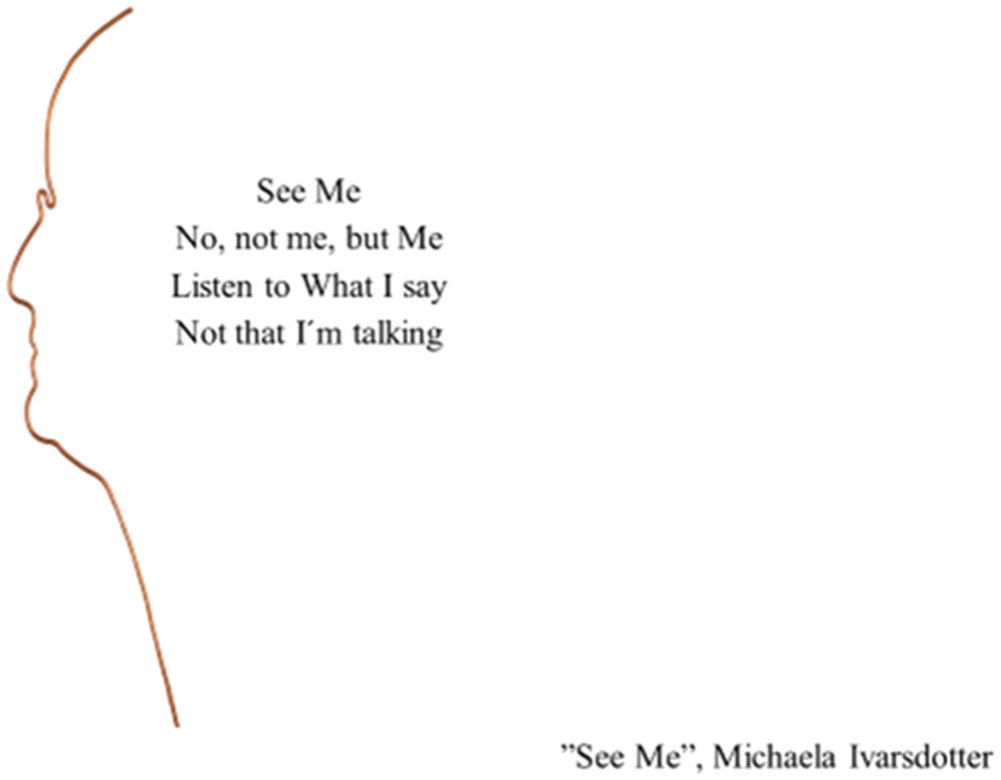
'See me'.

In this piece of art, titled 'See Me' (Figure 3), patients express the need to be seen and heard regardless of the situation—'See Me, No, not me, but Me, Listen to What I say, Not that I'm talking'. What happens within the professionals when they meet a patient? A sense of responsibility for the other person grows, not merely as a voluntary engagement, but as a demanding ethical call, where the right call of action is dictated by the present situation. Let's illustrate this with an example from the emergency care, highlighting Løgstrup's silent ethical demand.

Imagine working on an overcrowded ED, prioritising and sorting critically ill patients from noncritical ones who have to wait to see a doctor or be referred to another care level. In this situation, the ethical demand speaks to you. You see the patient's existential needs but are in a hurry and hesitate whether to do the more or not. You weigh the situation, thinking the critically ill are prioritised. Despite this, you feel an obligation to act and decide to hand over the noncritical patient's existential needs. This internal dialogue takes place within seconds, and you continue your work with critically ill patients. However, the silent ethical demand remains strong, and you keep thinking about the situation with the overcrowded ED when you come home: Was there anything else I should have done? Did they get the help they needed? Løgstrup might refer to this as a 'circulating manifestation', where your actions stem from your own situation and a more self‐centred mission with active reflection to determine what to do (Løgstrup 1992). This example could unfold differently if, upon passing by the noncritical patient you stop to assess whether there is something you could do to assist. Acting without consideration for being in a hurry or other personal concerns, could be stated as a spontaneous manifestation where you act without thinking of your own situation, a pre‐reflection (Løgstrup 1992).

According to the above‐described situation, Levinas takes the concept of the ethical demand even further, describing it as an imperative that you may not opt out of. Once you recognise the patient's needs and come close, you cannot simply turn around and leave. The ethical demand compels you to stay close, 'Face to Face' with the patient, and when you make eye contact, a sense of responsibility to act emerges. The closeness where you can see the other's vulnerability makes the ethical demand stronger. However, it is not the closeness itself that's in focus; Levinas uses the 'Face' as a metaphor for the otherness (Levinas 1993). The demand originates from the other and revolves around understanding them and allowing the other to decide what is best—not to renounce responsibility,—not to take over,—but to find balance.

As a professional your actions are not only based on the ethical demand; you also act according to your profession, which means that as a professional you ought to make the right choice, doing good for others, no matter the circumstance. Lavoie et al. (2006) describes this in terms of an asymmetrical relationship, a one‐way sense of duty. According to Delmar (2012), there is however, an imbalance of power in the relationship between patients and professionals, making every encounter ethically challenging with different barriers to a caring relationship. Moreover, each individual professional has to coordinate their actions with other professionals as well as the organisation (Holm 2001), were they may face external and/or internal obstacles. External obstacles could include efficiency demands, time pressure, technology, or the ethical climate in the workplace. Internal obstacles may encompass inner expectations, habits or bad habits, uncertainty, or lack of knowledge (Deschenes et al. 2020). Ethical decision‐making is inherently situational, and it is a challenge to truly grasp the meaning of patients' stories, which must be understood in the context of their real‐life experiences and 'lifeworld' (Greenfield and Jensen 2010).

## 5 Ethical Dilemma

In the workplace, there are diverse ethical dilemmas that often go unnoticed. These dilemmas may involve issues such as work overload impacting the quality of care (Haahr et al. 2020), concerns about respecting patient autonomy while avoiding harm (Jørgensen and Kollerup 2022), and the delicate balance between providing ethical care and navigating an unethical work culture. As a first responder at the scene of an accident, you are continuously moving between being and doing, and at the ED, between providing and responding. You may find yourself caught in an ethical dilemma, balancing the high demands of efficiency and time pressure with the patient's unmet existential needs and desire for a meaningful encounter (Elmqvist 2011; Elmqvist 2016).

### 5.1 Moral Sense Versus Moral Distress

**Figure 4:**
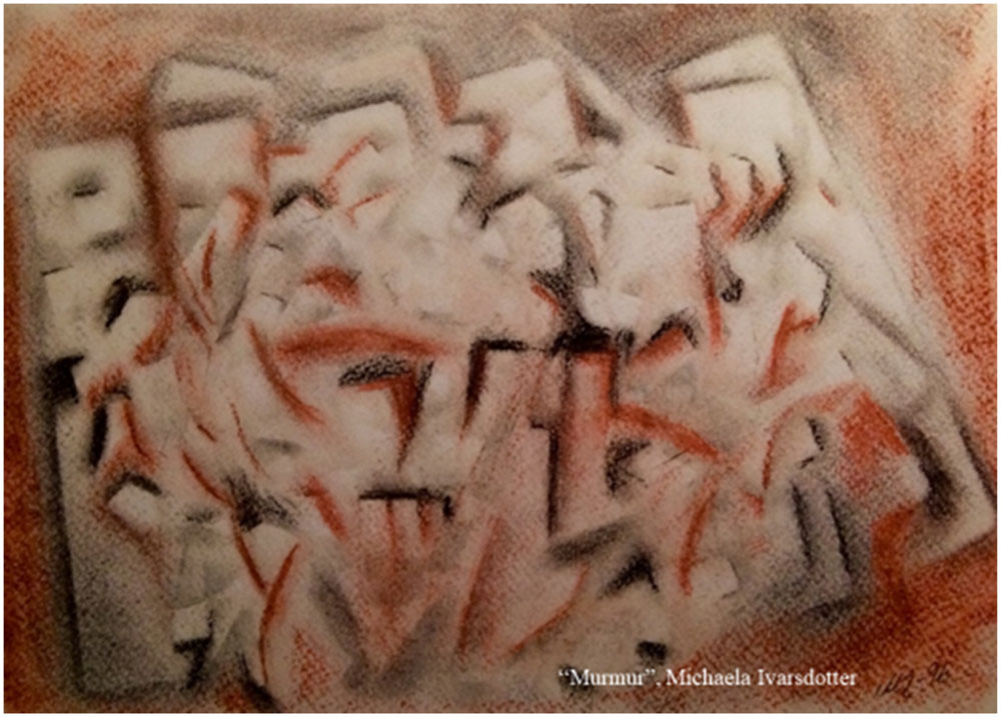
'Murmur'.

This piece of art, titled 'Murmur' (Figure 4), symbolises the inner turmoil, murmur in your soul, when one cannot follow the ethical demand and act as desired. Feelings of discomfort, such as powerlessness or inadequacy, may arise, leading to a bad conscience. The term 'moral distress' describes the stress experienced by healthcare providers in their daily practice (Deschenes et al. 2020). Jameton (1984) originally defined moral distress as a situation where one knows the right thing to do, but institutional constraints make it nearly impossible to pursue the right course of action’ (p.6). Moral distress can result in reduced job satisfaction, increased stress, and a higher risk of burnout symptoms among healthcare staff, ultimately affecting their ability to provide safe and high‐quality patient care. Despite its negative personal consequences, moral distress can also lead to positive outcomes, such as greater clarity and insight, fostering personal and professional growth (Deschenes et al. 2020). Therefore, these feelings can serve as a catalyst for reflection, enabling the development of a moral sense and ethical competence.

## 6 Ethical Stress

Ethical stress arises from ethical dilemmas where no solutions are truly satisfying (Manara et al. 2014). Sometimes, despite significant efforts, healthcare staff cannot alleviate patient's suffering, leading to shared experience of distress. In such situations, they become aware of their own bodies and emotions, leading to mutual experience of suffering (Morse et al. 2006). Constant exposure to suffering can emotionally drain staff, so they need to manage their emotional involvement to continue caring for other patients and avoid burnout. Instead of remaining in a problem‐solving mode, it is important to adapt our approach, and engage with the patient's perspective to find ways to cope (Manara et al. 2014). Ethical sensitivity, which involves being aware of and interpreting ethical issues, is crucial for navigating in these situations. According to Manara et al. (2014) ethical reflection can give the professionals opportunity to change their way of feeling or acting, and the relation between the professionals and the patient, the mutual exchange can help reduce healthcare staff's own suffering (Morse et al. 2006).

### 6.1 Compassion Versus Self‐Compassion

**Figure 5:**
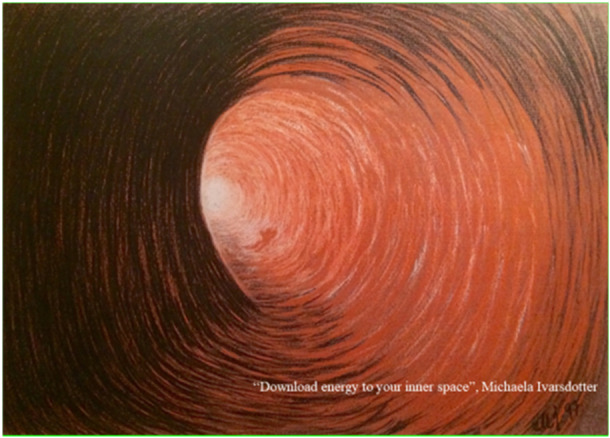
'Download energy to your inner space'.

This piece of art, 'Download Energy to Your Inner Space' (Figure 5), emphasises the importance of self‐care, learning how to take care of oneself and finding balance while caring for others. When professionals encounter patients’ needs and suffering, the compassion that arises in these interactions is an important catalyst. Compassion, defined as ‘a sensitivity to suffering in self and others, with a commitment to try to alleviate and prevent it’ (Gilbert 2014, p. 19), plays a crucial role in this context. It involves a commitment to alleviate, prevent and reduce suffering in both others and oneself (Gilbert 2014).

Compassion is a relational process that can be directed from oneself to another, from another to oneself, or from oneself to oneself as self‐compassion (Kirby et al. 2019). Often, healthcare providers focus on directing compassion towards others but may not be accustomed to practicing self‐compassion or receiving compassion from others. Learning to support oneself, practicing self‐compassion, and embracing compassion from patients can enhance well‐being and resilience in healthcare providers, ultimately contributing to increased job satisfaction (Dev et al. 2020). Being self‐compassionate becomes a way to download energy to your inner space. To address these challenges, it is important to foster support for ethical competence and encourage open discussions and reporting of ethical dilemmas (Albert et al. 2020).

## 7 Ethical Competence

Research on ethical competence has been limited, with the majority of studies focusing on the nurse's perspective (Poikkeus et al. 2014; Koskenvuori et al. 2019). A concept analysis shows that ethical competence can be defined in terms of character strength, ethical awareness, moral judgement skills and willingness to do good, which result in the best possible solutions for the patient and reduced moral distress at work (Kulju et al. 2016). Moreover, ethical competence requires feelings of compassion and concern for others (Pohling et al. 2016). Ethical competence, coupled with an emphatic approach, not only makes the patient feel cared for but also brings inner satisfaction from doing good. The emphatic approach involves a conscious presence close to the patient, providing both patients and professionals with existential support and a sense of meaning in a mutual space (Elmqvist 2011; Elmqvist 2016).

### 7.1 Give Versus Receive

**Figure 6:**
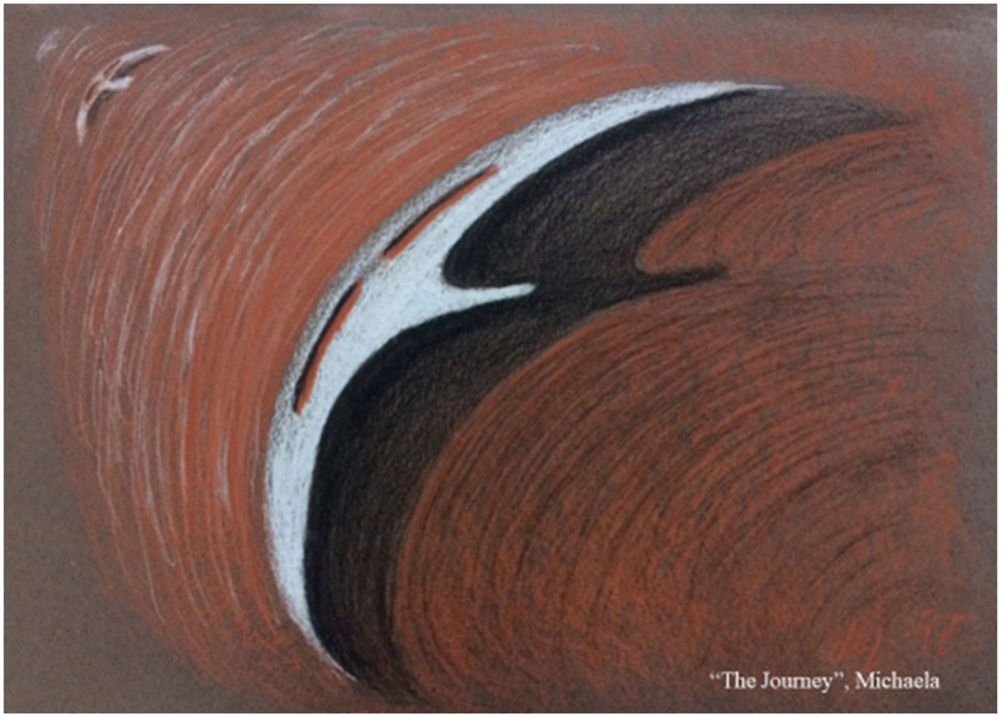
'The journey'.

This piece of art, titled 'The Journey' (Figure 6), symbolises the encounter—the common journey—between patients and the various professionals they meet within emergency care, emphasising the mutual trust involved in giving and receiving. According to Løgstrup, trust can be understood as a way of communicating; through communication, we give something and simultaneously receive something in return, creating an interdependence that Løgstrup calls trust (Holm 2001). When different lifeworld meets in the encounter within emergency care, language becomes the bridge connecting the patient's identity with the professional's identity. As professionals, we must learn the patient's language, relate to, and listen to their lifeworld. Treating the person as an active participant, not merely a passive recipient of care, involves allowing the patient express themselves, listening attentively, and learning from their experiences. We must provide clear explanations, addressing what, how, why, when, answering unspoken questions, and fostering a sense of predictability and meaning. Engaging in internal dialogue, questioning our own actions with self‐awareness, and challenging ourselves are crucial aspects of this process. Finally, we must ask each other questions and reflect together with other professionals to give and receive energy (Elmqvist 2011, 2016).

The concept of giving and receiving is connected to vulnerability. As Levinas emphasises, acting ethically means being open to others vulnerability, while recognising our own (Gjengedal et al. 2013). This involves daring to bear witness and being with others, to touch and dare to be touched (Naef 2006). Merleau Ponty poetically describes this as a simultaneous experience that happens when one hand touches another, the understanding of touching and being touched (or respond‐provide, give‐receive) (Merleau Ponty 1964/1992). Or as the wave when it breaks and curls over, it is the place in the wave where the water touches itself (Thomas 2005). Therefore, the encounter can be seen as a common journey of giving and receiving, creating a 'win‐win' situation, fostering a meaningful and transformative experience for all involved. Advocates help patients make their journey through the healthcare system as smooth as possible.

## 8 Discussion

Healthcare involves a variety of professions, and for the benefit of the patient, we must recognise the significance of professionals taking on the advocacy role from a caring science perspective, which includes the unique and shared experiences of the lifeworld. This is a challenge within the context of demanding efficiency and time pressure in emergency care. To address this, a concrete action plan for ethical reflections is needed to find a balance between giving and receiving, essential for healthcare professionals to avoid compassion fatigue. However, there are critical assessments regarding the challenges professionals face in acting independently due to the organisational structure, where each healthcare professional must align their actions with others and adhere to organisational boundaries. Examples demonstrate the dire consequences when healthcare organisations prioritise financial goals over the needs of the patients (Fotaki 2015). Health care systems sometimes disregard the patient's ability to participate in their care, forgetting that the system is composed of the people working within it (Mitchell and Bournes 2000). When organisations fail to provide safe and compassionate care, it becomes imperative to instill compassion at an organisational level (George 2018).

Maintaining the staff's ethical competence relies on a complex interplay between individual commitment and organisational support, primarily through education strategies and dedicated time for ethical reflection (Poikkeus et al. 2014; Kulju et al. 2016; Poikkeus et al. 2018; Andersson et al. 2022). In the context of ethical competence and the challenges faced by different healthcare professionals within emergency care, the Art of Caring model could be used for ethical reflections, as an approach to achieve a balance between patient advocacy, ethical considerations, and effective emergency care delivery. Achieving this goal will lead to better patient outcomes and a more supportive work environment for the entire emergency care team.

When viewing something as a figure against a background of something else, it becomes easier to reflect and uncover the essence of care that is, the Art of Caring. Concepts such as respond versus provide in situations when souls meet and souls separate, external versus internal obstacles when seeing the patient, moral sense versus moral distress as murmur in your soul, compassion versus self‐compassion to develop energy for patients and your own inner space, and finally give versus receive in a common journey within emergency care creates an awareness. In turn, an awareness of these concepts, will provide necessary tools for ethical reflections when practicing the Art of Caring model within emergency care.

## Conflicts of Interest

The authors declare no conflicts of interest.

## Data Availability

The data that support the findings of this study are available on request from the corresponding author. The data are not publicly available due to privacy or ethical restrictions.
